# Molecular interaction assays *in silico* of crotapotin from *Crotalus durissus terrificus* against the molecular target trypanothione reductase from *Leishmania braziliensis*

**DOI:** 10.1590/1678-9199-JVATITD-2024-0049

**Published:** 2025-04-04

**Authors:** Jamile Mariano Macedo, Mateus Farias Souza, Anderson Maciel Lima, Aleff Ferreira Francisco, Anderson Makoto Kayano, Maria Elisabeth Moreira de Lima Gusmão, Erika Crhistina Santos de Araújo, Guilherme Henrique Marchi Salvador, Marcos Roberto de Mattos Fontes, Juliana Pavan Zuliani, Andreimar Martins Soares

**Affiliations:** 1 Federal Institute of Rondônia, Porto Velho Calama Campus, Porto Velho, RO, Brazil.; 2 Laboratory of Protein Biotechnology and Education Applied to One Health, Oswaldo Cruz Foundation, FIOCRUZ Rondônia, Porto Velho, RO, Brazil.; 3 Postgraduate Program in Biodiversity and Biotechnology - BIONORTE Network (PPGBIONORTE), Federal University of Pará, Belém, PA, Brazil.; 4 International Network of Research and Excellence Knowledge of the Western Amazon (RED-CONEXAO), Porto Velho, RO, Brazil.; 5 Tropical Medicine Research Center (CEPEM/SESAU-RO), Porto Velho, RO, Brazil.; 6 Postgraduate Program in Cellular and Molecular Biology, Oswaldo Cruz Institute, Oswaldo Cruz Foundation (Fiocruz), Rio de Janeiro, RJ, Brazil.; 7 Laboratory of Cellular Immunology Applied to Health, Oswaldo Cruz Foundation, FIOCRUZ Rondônia, Porto Velho, RO, Brazil.; 8 Department of Biophysics and Pharmacology, Institute of Biosciences, São Paulo State University (UNESP), Botucatu, SP, Brazil.; 9 Institute for Advanced Studies of the Sea (IEAMar), São Paulo State University (UNESP), São Vicente, SP, Brazil.; 10 National Institute of Science and Technology of Epidemiology of the Western Amazon (INCT EpiAmO), Porto Velho, RO, Brazil.

**Keywords:** Snake venoms, Inhibition, Neglected tropical diseases, Molecular dynamics, Recombinant expression

## Abstract

**Background:**

Leishmaniasis is a neglected disease that mainly affects impoverished populations and receives limited attention from governments and research institutions. Current treatments are based on antimonial therapies, which present high toxicity and cause significant side effects, such as cardiotoxicity and hepatotoxicity. This study proposes using crotapotin, isolated from *Crotalus durissus terrificus* venom, as a potential inhibitor of the enzyme trypanothione reductase from *Leishmania braziliensis* (LbTR).

**Methods:**

*In silico* assays were conducted to evaluate the interaction of crotapotin with LbTR using molecular docking and molecular dynamics techniques. Recombinant LbTR was expressed in *E. coli*, and its enzymatic activity was confirmed. The inhibitory action of crotapotin on LbTR was then tested in enzymatic assays.

**Results:**

The stability of these interactions was confirmed over 200 ns molecular dynamics simulations, with a clustering analysis using the GROMACS method revealing a total of 12 distinct clusters. The five most representative clusters showed low RMSD values, indicating high structural stability of the LbTR-crotapotin complex. In particular, cluster 1, with 3,398 frames and an average RMSD of 0.189 nm from the centroid, suggests a dominant stable conformation of the complex. Additional clusters maintained average RMSD values between 0.173 nm and 0.193 nm, further reinforcing the robustness of the complex under physiological conditions. Recombinant LbTR expression was successful, yielding 4.8 mg/L with high purity, as verified by SDS-PAGE. In the enzymatic assays, crotapotin partially inhibited LbTR activity, with an IC_50_ of 223.4 μM.

**Conclusion:**

The *in silico* findings suggest a stable and structured interaction between crotapotin and LbTR, with low structural fluctuation, although the inhibition observed in *in vitro* assays was moderate. These results indicate the potential of crotapotin as a promising basis for developing specific LbTR inhibitors, contributing to the bioprospecting of new antiparasitic agents.

## Background

Leishmaniasis affects approximately 12 million people, primarily distributed across South Asia, Sub-Saharan Africa, India, Latin America, and the Caribbean, with 2 million cases of cutaneous leishmaniasis (CL) and between 50,000 to 90,000 cases of visceral leishmaniasis (VL) annually. It is estimated that over 350 million people are susceptible to infection, largely due to unfavorable environmental conditions [1, 2]. *Leishmania* (Phylum: Euglenozoa; Order: Kinetoplastea; Family: Trypanosomatidae), transmitted by phlebotomine sandflies of the genera *Phlebotomus* and *Lutzomyia*, infects mammals when promastigote forms of the parasite are introduced during a bite, subsequently multiplying in phagocytic cells as amastigotes [3-5].

CL is the most common form, especially in the Americas, the Mediterranean, the Middle East, and Central Asia [6]. In contrast, VL is more severe and lethal, with 73% of cases occurring in countries like India and Brazil [7]. *Leishmania* drug resistance poses a growing challenge, involving genetic mutations that reduce the efficacy of available treatments, such as sodium stibogluconate, meglumine antimoniate, liposomal amphotericin B, miltefosine, paromomycin, and pentamidine, which exhibit a cure rate of approximately 90%. However, efficacy varies by species and region [8-11].

Recent studies highlight the toxicity of these treatments, including cardiac and hepatic insufficiencies, as well as contraindications in elderly and pregnant individuals, which underscores the need for new therapies [12-15]. In this context, the development of new antiparasitic agents has focused on active compounds from medicinal plants, drug repurposing, and the identification of metabolic targets in the parasite [16, 17]. Drugs such as pentamidine and amphotericin B, initially used for fungal infections, and miltefosine, developed for breast cancer, have shown promise in repurposing for VL treatment [18-20].

Recent studies on snake venoms have shown promising effects against parasites, opening new therapeutic possibilities. Mendes et al. [21] investigated peptides derived from phospholipases A_2_ (PLA_2_) and oligoarginines, observing increased membrane permeability in parasites, particularly in promastigotes and amastigotes of *Leishmania* spp. Modified peptides, such as p-AclR7, demonstrated effective antiparasitic activity and low cytotoxicity for host cells, making them potential candidates for safe and targeted antileishmanial therapies [21]. Other studies on molecules with leishmanicidal activity, such as antimicrobial peptides (AMPs), reveal their potential in various therapeutic approaches. AMPs, primarily known for their antibacterial activities [22], have also been explored for their action against *Leishmania* spp., demonstrating effective antiparasitic properties [23, 24]. Moreover, bioactive peptides from diverse natural sources have shown dual activities, acting simultaneously against bacteria and parasites, enhancing their therapeutic value in co-infected or mixed infections [25-27]. Borges et al. [28] conducted a study evaluating the toxin BnSP-7, a phospholipase A_2_ (PLA_2_) Lys49 from *Bothrops pauloensis* venom, for its antiparasitic effects against *Toxoplasma gondii*. BnSP-7 demonstrated the ability to reduce tachyzoite adhesion and proliferation, showing low cytotoxicity in HeLa cells and modulating the immune response of host cells, highlighting its potential as a candidate for the development of targeted antiparasitic therapies [28]. These studies underline the potential of snake venom peptides as promising antiparasitic agents.

**Figure 1.**
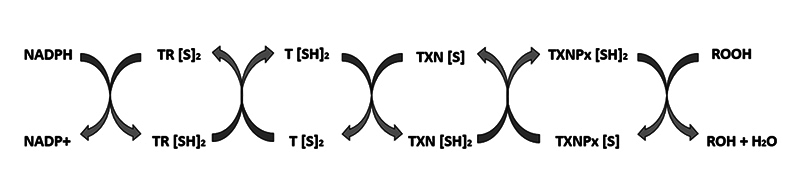
Schematic representation of the trypanothione-dependent redox pathway in parasites. The enzyme trypanothione reductase (TR) reduces oxidized trypanothione (T[S]_2_) to its dithiol form (T[SH]_2_) using NADPH as an electron donor. The reduced trypanothione then transfers electrons to thioredoxin (TXN[S]), which, in its reduced form (TXN[SH]_2_), activates thioredoxin peroxidase (TXNPx[SH]_2_). The reduced TXNP[S] converts peroxides (ROOH) into alcohol (ROH) and water (H_2_O), protecting the parasite from oxidative stress. Adapted from: Hamilton et al. [36].

In this context, trypanothione reductase (TR) is an enzyme that functions as an intracellular antioxidant in trypanosomatids, neutralizing reactive oxygen species (ROS) and protecting cells against oxidative damage. While TR shares functional similarities with glutathione reductase (GR), an enzyme ubiquitous in eukaryotes (animals, plants, yeasts) and some bacteria, key distinctions exist. Both enzymes are homodimeric, possess a molecular mass of approximately 54 kDa, utilize FAD as a cofactor, and exhibit specificity for NADPH as an electron donor. However, TR uniquely catalyzes the reduction of oxidized trypanothione (TS2) to its reduced form (T(SH)2), employing NADPH as electron donor (Figure 1) [29-35].

The presence of TR and GR in different organisms makes the former an intriguing target for drug development against trypanosomatid parasites, as it has no homologs in the human host. TR and GR share some structural similarities, such as the presence of FAD and NADPH binding domains and a dimeric structure. However, there are critical structural differences that make TR a unique pharmacological target [30]. TR has an additional domain responsible for binding trypanothione, a glutathione dimer specific to trypanosomatids, whereas GR lacks this domain and binds only monomeric glutathione [31, 34]. Furthermore, TR has a specific arrangement of amino acid residues in its active site that differs from GR, allowing for highly specific binding and reduction of trypanothione [35]. These structural differences not only confer substrate specificity to TR but also enable the design of inhibitors that can selectively block TR activity without affecting the glutathione-based redox system in host cells [37-39]. 

TR is vital for the survival of trypanosomatids, as these parasites rely on this enzyme to maintain their internal redox environment balanced and to protect themselves against oxidative stress generated by the host’s immune system. The absence of a homologous enzyme in humans enables the development of selective drugs, minimizing side effects and toxicity in patients [30, 31, 34]. For these reasons, TR is considered a validated target for the design of new trypanocidal agents, representing a crucial focus in the search for effective treatments against leishmaniasis.

In this context, snake venoms emerge as potential sources of new therapeutic compounds. Due to their complexity and the presence of various toxins, these venoms have been studied for their pathophysiological effects and potential for drug development [40-42]. Abdullahi et al. [43] conducted a systematic review on the antiprotozoal effects of snake venoms and their fractions, highlighting the efficacy of crude venoms and phospholipase A_2_ (PLA_2_) isoforms against protozoa. The review found promising evidence of synergism, especially with PLA_2_ from *Bothrops asper*, and identified a need for further studies on other venom components, such as metalloproteinases, serine proteases, and three-finger toxins, whose antiprotozoal actions remain underexplored. The systematic review by Almeida et al. [44] identified several studies that underscore snake venoms as sources of molecules with antiparasitic activity against malaria and trypanosomatids. Promising results were found involving phospholipases A_2_, metalloproteases, and lectins, highlighting the potential of these compounds in combating medically relevant protozoa [44]. *Crotalus durissus terrificus* (South American rattlesnake) venom displays a remarkable protein profile, including convulxin, gyroxin, crotoxin (and its subunits) and crotamine, each with well-documented specific biological activities, such as neurotoxic, cytotoxic and immunomodulatory properties [45-47].

Crotoxin, the main neurotoxin in the venom, is composed of a toxic phospholipase A_2_ (PLA_2_) subunit and crotapotin (CA), an acidic subunit essential for the structural and functional stability of PLA_2_ [48, 49]. Unlike PLA_2_, crotapotin, which acts as a molecular chaperone facilitating the folding and active conformation of PLA_2_, remains underexplored in terms of its pharmacological potential. While PLA_2_ is recognized for its cytotoxic activity and immunomodulatory properties, with anti-inflammatory and immunosuppressive effects [45, 47], crotapotin, due to its lack of intrinsic toxicity, presents a unique perspective as a candidate for therapeutic investigations, focusing on safety and immune modulation [50,51].

Recent studies indicate that crotapotin not only stabilizes PLA_2_ but may also influence the immune response, acting through anti-inflammatory and immunosuppressive mechanisms, with unexplored potential for the development of safe antiparasitic agents [50,51]. In contrast to PLA_2_, which has been extensively studied for its anti-trypanosomatid and antileishmanial activities [52], crotapotin remains under-investigated, particularly regarding its application in inhibiting critical enzymatic targets, such as trypanothione reductase (TR). The present study aims to address this gap by evaluating crotapotin from *C. d. terrificus* venom against TR from *Leishmania braziliensis* as a potential antiparasitic agent.

## METHODS

### Crotapotin purification

The venom from *Crotalus durissus terrificus* used in this study was obtained from the venom bank of the Center for the Study of Biomolecules Applied to Health, affiliated with the Oswaldo Cruz Foundation and the Federal University of Rondônia - CEBio/FIOCRUZ-RO/UNIR. The activity related to access to Genetic Heritage was registered in SisGen, in compliance with Law n. 13.123/2015 and its regulations: CGEN/CNPq 010627/2011-1; IBAMA 27131-2 and CEBio UNIR-FIOCRUZ-RO (register CGEN A4D12CB and IBAMA/SISBIO 64385-1).

Replicates of 10 mg of the dried venom pool were solubilized in 1 mL of 50 mM ammonium formate, pH 3.5, and centrifuged at 755 × g for 10 minutes. The supernatant was applied to a Sephadex G-75 column (10 × 300 mm) equilibrated with the same dilution buffer, at a flow rate of 0.8 mL/minute. Fraction elution was monitored at an absorbance of 280 nm using an Akta Purifier 10^®^ chromatography system (GE Lifescience Healthcare), collected, and stored in a freezer at -20 ºC. After drying, the fraction labeled F3, obtained from size exclusion chromatography, was solubilized in 0.1% trifluoroacetic acid (TFA) (solution A) and subjected to high-performance liquid chromatography (HPLC) on a C-18 column (25 mm × 4.6 mm, Supelco), previously equilibrated with solution A and eluted under a 0 to 70% gradient of solution B (99.9% acetonitrile and 0.1% TFA) in 5 column volumes, at a flow rate of 1 mL/min, in an Akta Purifier 10® chromatography system (GE Lifescience Healthcare). Elution was monitored at 280 nm. The fraction obtained from reverse-phase chromatography was dried and rechromatographed on DEAE-Sepharose (10 x 350 mm) in 50 mM ammonium bicarbonate buffer at pH 8.0.

For protein quantification, the assay was based on the Lowry method [53], using the BIO-RAD assay kit. The methodology was previously described by Laemmli [54].

### Protein identification

A sample amount of approximately 1.4 mg was dissolved in 100 µL of a solution containing 70% acetonitrile and 30% 1% TFA (v/v). For MALDI-TOF MS analysis, 2 µL of this prepared sample solution was spotted directly onto a MALDI plate. The spotted sample was then allowed to dry completely at room temperature. Following this, a 2 µL aliquot of a saturated solution of the ionizing matrix, α-cyano-4-hydroxycinnamic acid, dissolved in a 50:50 (v/v) mixture of acetonitrile and 0.1% TFA, was overlaid onto the dried sample spot. This final mixture was also allowed to dry at room temperature before analysis.The sample preparations were conducted in triplicate. For equipment calibration, a standard protein mix ranging from 700 Da to 37000 Da was used. Once the samples with the matrix were dry, the plate was inserted into the Autoflex instrument, a TOF/TOF mass spectrometer manufactured by BRUKER. FlexControl software was employed for the analysis, and flexAnalysis software was utilized for data processing. For data acquisition, FlexControl was initially configured to detect a mass range from 1 kDa to 20 kDa, corresponding to the molecular masses of the proteins in the sample. Mass spectra were acquired in positive linear mode, with 10,000 spectra collected per analyzed sample. Matrix suppression was applied for masses below 1 kDa. The equipment settings were as follows: ion source 1 at 19.50 kV, ion source 2 at 18.20 kV, and lens at 7 kV. Laser energy was maintained between 70-85%. For analysis of the spectra via flexAnalysis software, ions within the range of 1 kDa to 20 kDa were observed.

### *In silico* assays

In this study, the theoretical structural model of trypanothione reductase (TR) from *Leishmania braziliensis* (UniProt ID: A4H480) in complex with crotapotine (Uniprot ID: 3R0L) was generated using the AlphaFold 3 webserver [55]. 

Since the simulations were performed with the homodimeric LbTR enzyme in its native form, incorporating NADPH, FAD, and T[S]_2_ molecules into their respective binding sites was essential. The system setup was conducted using the CHARMM-GUI tool, positioning the LbTR within a simulation box with a 1.5 nm clearance from each edge, taking the furthest atom on each Cartesian axis as a reference. This distance ensures that the minimum distance between the molecules and their periodic images exceeds the cutoff for Verlet interactions (1.2 nm). The generated box was explicitly solvated using the TIP3P water model. The system’s pH was adjusted to 7.4, based on the protonation states of ionizable residues and His tautomers calculated via the APBS web server. To neutralize the system, some water molecules were substituted with positive (Na^+^) and negative (Cl^-^) ions, randomly distributed within the box to achieve an ionic strength of 150 mM. Bonds involving hydrogen atoms were constrained using the LINCS algorithm. Additionally, simulations incorporated hydrogen mass repartitioning (HMR), allowing for an integration time step of up to 4 fs [56, 57].

Molecular dynamics (MD) simulations were performed using GROMACS [56] and the CHARMM36m force field [58]. Next, energy minimization was done using the Steepest Descent algorithm to eliminate inconsistent contacts and refine the solvation layer on the protein’s solvent-accessible surface, reducing the system’s potential energy to below 1000 kJ/mol/nm. The system was then equilibrated under an isochoric-isothermal ensemble (NVT) for 1 ns, generating velocities according to the Maxwell-Boltzmann distribution with the V-Rescale thermostat. This was followed by equilibration under an isothermal-isobaric ensemble (NPT) using the Berendsen barostat at 1 bar [59]. 

Subsequently, unrestricted duplicates of 100 ns simulations were conducted using the Nose-Hoover thermostat [60, 61]. Non-bonded interactions were calculated within a 12 Å radius, utilizing a switching function between 10 and 12 Å. Long-range electrostatic interactions were managed using the particle-mesh Ewald method. The simulation was maintained at a temperature of 310.15 K and a pressure of 1 bar.

Post-simulation, the trajectory was analyzed to extract measurements of the root mean square deviation (RMSD) of the main chain to assess stability. Additionally, a refined categorization of the trajectory was achieved through the GROMOS clustering method [62]. This method was based on an RMSD threshold of 2 Å to effectively group similar conformations, balancing the need for detailed differentiation of clusters while avoiding overly fragmented categorization, thereby ensuring a comprehensive yet discerning analysis of the trajectory data.

### Recombinant expression and characterization of *Leishmania braziliensis* trypanothione reductase

The *L. braziliensis* trypanothione reductase gene [chromosome: 5; NC_009298.2 (109589.111064)] was inserted into the pET28a(+) expression vector, obtained from Biofast^®^, containing a poly-histidine tag and restriction enzyme sites for NcoI and XhoI, necessary for subcloning. For expression, the plasmid was transformed into electrocompetent *E. coli* BL21DE3 and selected on solid medium containing kanamycin (25 µg/mL) after 12 hours of incubation at 37 ºC. The steps from recombinant expression to obtaining the bacterial lysate were performed as described by Sambrook and Russel [63].

The supernatant was filtered through a 0.45 µm membrane and subjected to purification by affinity chromatography using immobilized Ni²⁺ resin, with buffered systems (20 mM TRIS, pH 8.0) containing different concentrations of imidazole (10 mM for column equilibration, 30 mM for washing, and 500 mM for elution) at a flow rate of 1 mL/min, monitored at 280 nm. Protein concentration was determined as described in section “Crotapotin purification”. The collected fraction was subjected to 12.5% polyacrylamide gel electrophoresis (SDS-PAGE) under reducing conditions.

### Enzymatic activity and kinetic constant determination of recombinant LbTR

The enzymatic activity of LbTR was analyzed spectrophotometrically as previously described [36]. The change in absorbance over time (ΔA/min) was determined from the first 60 seconds of the reaction course. The increase in absorbance at 412 nm was used to determine substrate consumption as a measure of enzymatic activity, calculated using the molar extinction coefficient of 2-nitro-5-thiobenzoic acid (TNB), ε = 13.6 mM⁻¹ × cm⁻¹ (equation 1). The production of two TNB molecules for each T[SH]₂ was considered in calculating the initial velocities (equation 2). This approach enabled the determination of *K*_*m*_ and *V*_*max*_ by non-linear regression fitting of the data to Michaelis-Menten kinetics, according to the following equations [64].

$$V_{0}=\;\frac{∆A}{\varepsilon l}$$ (1)

*V*_*0*_: reaction rate 

*ΔA*: absorbance changes at 412 nm

*ε*: molar extinction coefficient of the chromophore TNB

*l*: optical path length (1 cm)

$$V_{0}^{{T[S]}_{2}}=V_{0}^{TNB}{\times 2}^{-1}$$ (2)

*V*_*0*_ *T[S]*_*2*_: actual reaction rate adjusted for substrate T[S]_2_

*V*_*0*_ *TNB*: reaction rate for the chromophore TNB

$$V_{0}=\frac{V_{max}\left[S\right]}{K_{m}+[S]}$$ (3)

*V*_*max*_: maximum reaction rate

*K*_*m*_: Michaelis-Menten constant

[*S*]: substrate concentration

The assays were performed in 96-well plates using a final volume of 300 µL containing the enzyme at 0.04 µM in the presence of 40 mM HEPES buffer and 1 mM EDTA at pH 7.5, 0.15 mM NADPH, < 0.1% DMSO, 25 µM DTNB, and T[S]₂ at concentrations of 60, 50, 40, 30, 20, 10, and 1 µM. The reaction was pre-incubated at 28 °C for 5 minutes before being initiated by the addition of T[S]₂. Enzyme activity was measured over 20 minutes using an Eon Biotek^®^ spectrophotometer at 28 °C. All reagents used in this study were purchased from SIGMA-Aldrich^®^. The analysis was replicated in two independent experiments, and the mean absorbances were used to determine initial rates and kinetic constants. Graphs were plotted using GraphPad Prism 9.4 and Excel 2021.

### Evaluation of the inhibitory effect of crotapotin

The calculation of the inhibition percentage was performed using the following equation:

$$100\times \left(\frac{a}{b}\right)=Activity\;\left(\%\right)$$ (4)

where “a” represents the absorbance value in the presence of the test compound, and “b” represents the absorbance value in the absence of the test compound. The inhibitory activity of crotapotin on LbTR was determined by measuring the half-maximal inhibitory concentration (IC₅₀). IC₅₀ values were calculated using the non-linear regression model available in GraphPad Prism 9.4.

$$y=\frac{100}{\left(\frac{1+x}{{IC}_{50}}\right)}$$ (5)

The enzyme inhibition assay was conducted by pre-incubating the enzyme with the toxins at various concentrations, from 100 μM to 200 μM, for 5 minutes before the addition of T[S]₂. The control, without any inhibitor, was considered as 100% enzymatic activity. Reaction conditions were maintained in a final volume of 300 µL containing the enzyme (0.04 µM) in the presence of 40 mM HEPES buffer and 1 mM EDTA at pH 7.5, 0.15 mM NADPH, 25 µM DTNB, < 0.1% DMSO, and 1 µM T[S]₂. The increase in absorbance at 412 nm was monitored over a 20-minute period at 28 °C.

## RESULTS

### Prediction of crotapotin-mediated inhibition of LbTR

**Figure 2.**
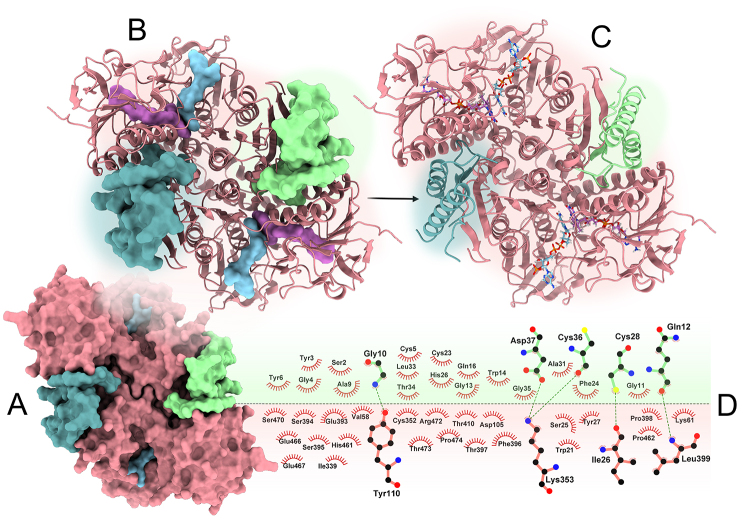
Predicted binding of crotapotin to *Leishmania braziliensis* trypanothione reductase (LbTR). The model depicts LbTR (salmon) as a homodimer with crotapotin (pale green and cadet blue) bound at both active sites. **(A)** Surface representation showing crotapotin’s spatial positioning relative to LbTR’s active sites. **(B)** Ribbon model of the LbTR dimer with FAD, NAD, and crotapotin represented as surfaces, indicating potential substrate access obstruction. **(C)** Ribbon model of the LbTR dimer complexed with FAD, NAD, and crotapotin. **(D)** Interaction map highlighting key residues in the LbTR-crotapotin interface, with hydrogen bonds depicted as green dotted lines and hydrophobic interactions as red protrusions.

The generated theoretical model predicts that crotapotin, the non-enzymatic component of the crotoxin complex, can interact with *Leishmania braziliensis* trypanothione reductase (LbTR) in a manner that disrupts its enzymatic function. Utilizing AlphaFold 3, a high-confidence structural model of the LbTR-crotapotin complex was generated, incorporating essential cofactors FAD and NADPH to capture a realistic representation of LbTR in its active, homodimeric state. The model reveals that crotapotin forms multiple interactions with residues involved in substrate binding and catalysis (Figure 2). These interactions indicate that crotapotin can occupy the binding pocket of the enzyme’s active sites, potentially acting as a steric inhibitor. Thus, crotapotin could obstruct substrate access to the active sites or induce conformational alterations that impair catalytic function, thereby inhibiting LbTR activity.

High-confidence scores (pLDDT > 90) for regions involved in the LbTR-crotapotin interaction lend support to this proposed inhibitory mechanism. The predicted binding orientation of crotapotin within this pocket is further stabilized by hydrogen bonds and hydrophobic contacts (Figure 1), which are likely to enhance its binding affinity to LbTR. Analysis of the interaction map suggests that crotapotin’s binding involves not only surface-level contacts but also penetrates into the enzyme’s active region, indicating a targeted binding mode that may selectively inhibit LbTR without interacting with unrelated sites.

The molecular dynamics (MD) simulation of the LbTR in complex with crotapotin, conducted in duplicates over 100 ns, was evaluated to assess the stability of the interaction and the conformational landscape of the complex. Root mean square deviation (RMSD) analyses of crotapotin-A, crotapotin-B, and LbTR were used as primary indicators of stability, and clustering analysis was employed to identify the predominant structural conformations of the complex.

**Figure 3.**
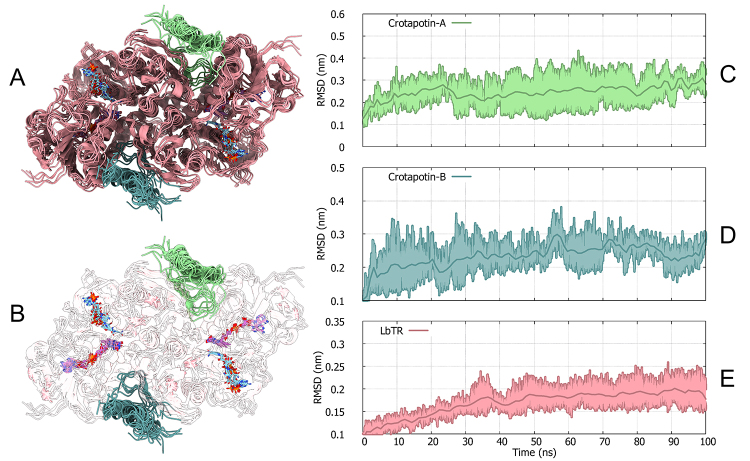
Stability and conformational analysis of the LbTR-crotapotin complex. **(A)** Ribbon model of LbTR (salmon) with two crotapotin molecules (pale green and cadet blue) bound at the active sites. The five most populated cluster centers, extracted from the concatenated 100 ns trajectory duplicates, are superimposed to illustrate LbTR’s conformational diversity and crotapotin’s structural behavior, along with **(B)** LbTR cofactors FAD and NADPH. **(C)** RMSD profiles showing the structural stability of crotapotin-A, **(D)** crotapotin-B (cadet blue), and **(E)** LbTR (salmon). RMSD values indicate that crotapotin stabilizes around 0.3 nm and LbTR around 0.25 nm, supporting stable binding and minimal conformational shifts throughout the simulation period. Each graph highlights the RMSD curves for both duplicates (sidelines) plotted around their respective mean values (center lines), with colors corresponding to the legend in each graph.

The RMSD profiles (Figure 3) indicate stable binding of both crotapotin monomers (A and B) to LbTR throughout the simulation. Crotapotin-A and crotapotin-B exhibited average RMSD values around 0.3 nm, with only minor fluctuations, suggesting stable engagement with the active site regions of LbTR. LbTR itself maintained an RMSD close to 0.25 nm, highlighting the structural integrity of the enzyme in the presence of bound crotapotin. These results imply that crotapotin does not induce significant structural distortions within LbTR, reinforcing its potential as a stable binding partner.

To further characterize the structural dynamics, a clustering analysis was performed on the concatenated 200 ns simulation trajectory using the GROMOS clustering method, resulting in a total of 12 distinct clusters. The five most populated clusters are displayed in the left panel of the figure, representing the central structures for each of these conformational states. Cluster 1 was the most populated, comprising 3,398 frames with an average RMSD of 0.189 nm from the centroid structure, suggesting that this cluster represents the dominant stable conformation of the LbTR-crotapotin complex. Clusters 2 and 3, with 3,384 and 1,427 frames respectively, also maintained low average RMSD values (0.193 nm and 0.183 nm), indicating high structural similarity and stability among the conformations sampled during the simulation.

Clusters 4 and 5, while less populated, also displayed relatively low RMSD values (0.182 nm and 0.173 nm), contributing additional stable configurations to the complex’s conformational ensemble. The presence of these closely related clusters with minor RMSD variations underscores the structural stability and resilience of the LbTR-crotapotin interaction under physiological conditions.

The distribution and stability of these clusters suggest that the LbTR-crotapotin complex predominantly exists in a few highly similar conformations, with crotapotin consistently bound in a manner that is unlikely to disrupt LbTR’s overall structure. This stable binding suggests that crotapotin effectively associates with LbTR’s active sites, potentially acting as a conformationally stable inhibitor.

### Crotapotin purification

**Figure 4.**
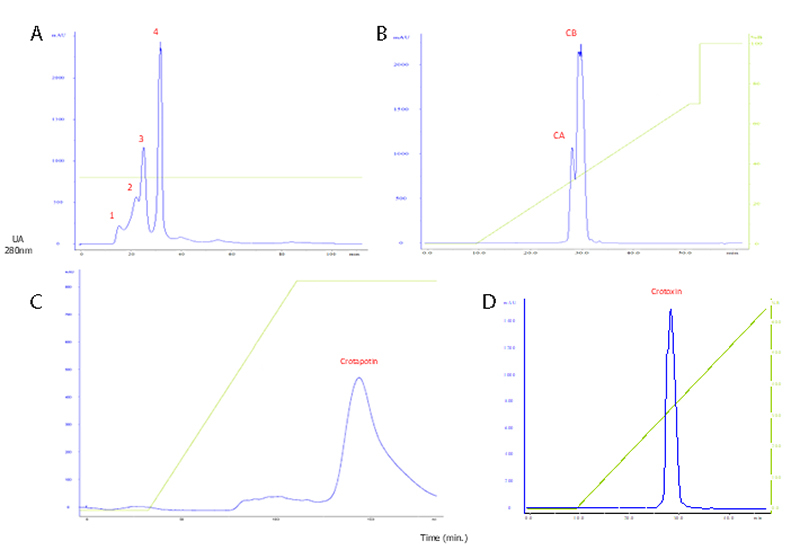
The figures display the chromatographic fractionation profiles for obtaining crotapotin. On the X-axis is time measured in minutes; on the right Y-axis, the concentration of eluent B; and on the left Y-axis, optical density expressed in AU (280 nm). In the inset, SDS-PAGE of the fractions after RP-HPLC. **(A)** Chromatographic profile of the *C. d. terrificus* venom pool, showing four fractions labeled a1 to a4. The third peak (A3) was suggestive of crotoxin. **(B)** Profile of fraction A3 re-chromatographed on a C18 column. The CA and CB subunits were not isolated. **(C)** The fraction was re-chromatographed on a DEAE Sepharose column, where a single peak suggestive of crotapotin was observed. **(D)** Profile suggestive of crotapotin after re-chromatography on a C18 column.

For the purification of crotapotin, a chromatographic sequence was employed, starting with size-exclusion chromatography on Superdex G75 resin. Four fractions were observed upon elution, as described in the literature, with the third peak suggestive of crotoxin. This third fraction was re-chromatographed on a reverse-phase column; however, unlike the profile described in the literature, no separation between crotapotin and PLA_2_ was observed. This fraction was subsequently re-chromatographed on a DEAE Sepharose column, where separation of crotapotin was achieved. In the fourth step, the lyophilized fraction was re-chromatographed on a C18 column. Figure 4 shows the chromatographic profiles obtained at each stage.

**Figure 5.**
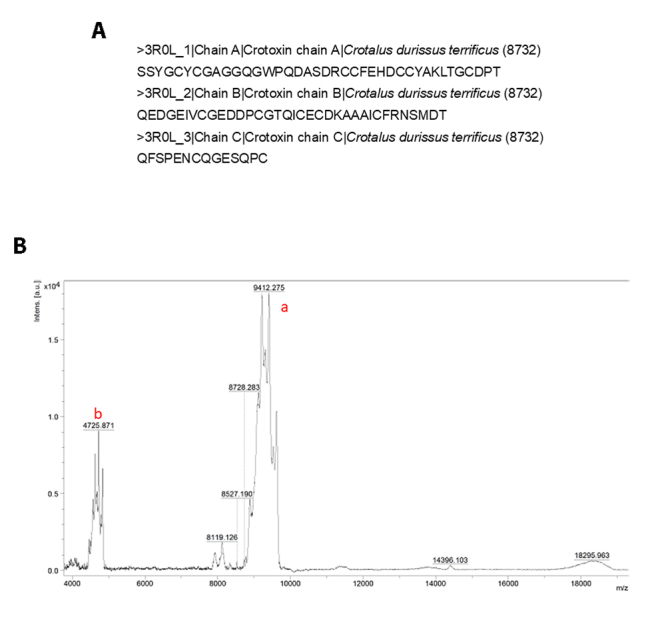
Molecular characterization of crotapotin from *C. d. terrificus* using mass spectrometry.

The identity of the isolated protein as crotapotin was confirmed by MALDI-TOF MS analysis. Using α-cyano-4-hydroxycinnamic acid as the matrix, a chromatogram (Figure 5 A ) was obtained, and its corresponding mass spectrum (Figure 5 B ) revealed a major peak at *m/z* ~9412, consistent with the expected mass of crotapotin, as well as a doubly charged ion at *m/z* ~4735. Combined, these data provide further support for the identification of the analyzed protein as crotapotin.

### Recombinant expression, purification, and characterization of LbTR

**Figure 6.**
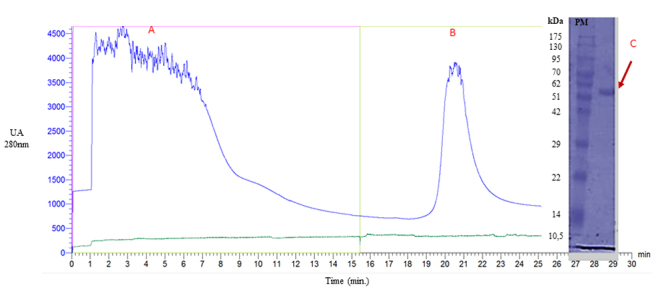
Chromatographic profile of LbTR purification. Chromatographic profile corresponding to the fractionation of the recombinant expression product from Ni-NTA affinity chromatography after 4 hours of induction. On the X-axis, time measured in minutes is displayed, and on the left Y-axis, optical density expressed in AU (280 nm). Fraction A (0-16 min) represents material with no affinity for the resin. Fraction LbTR (19-23 min) represents the material eluted after increasing the imidazole concentration (500 mM imidazole). Electrophoretic profile of the affinity-purified protein under reducing conditions. The arrow indicates the 54 kDa protein.

The product of bacterial expression, after appropriate processing (centrifugation and lysis), was subjected to procedures to obtain the recombinant protein of interest. The inserted polyhistidine tail, expressed contiguous to the protein, allows purification using techniques based on divalent cations (Ni²⁺ or Co²⁺). In this context, nickel Ni-NTA affinity chromatography was applied in the present work. As a result, two fractions were observed: the first, consisting of all material that showed no interaction with the stationary phase, defined as “bacterial lysate” (6A); and the second, designated as LbTR, consisting of material retained in the column and eluted upon the addition of 100% elution buffer containing a high concentration of imidazole, which competes for the metal ion, displacing the recombinant protein (Figure 6).

The electrophoretic analysis (SDS-PAGE) of the fractions obtained during the purification process showed a protein band with a relative molecular mass of approximately 54 kDa, corresponds to peak B, corresponding to its monomeric form, a value similar to that reported in the literature for other TRs [65-68] and compatible with the theoretical values obtained from the amino acid residues, totaling 53,381.23 Da (https://web.expasy.org/cgi-bin/protparam/protparam).

**Figure 7.**
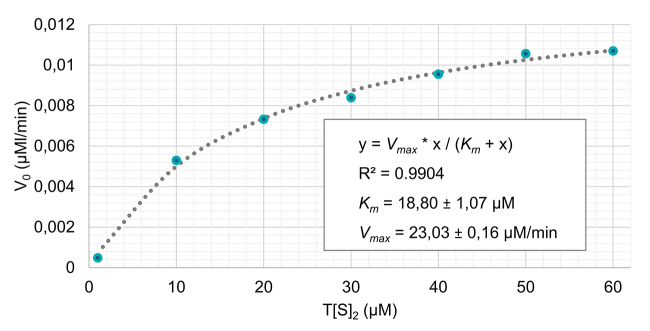
Enzymatic kinetics of the reduction of T[S]₂ to T[SH]₂ by the catalytic action of LbTR. The enzyme concentration was fixed at 0.04 µM, while substrate concentrations varied from 1 to 60 µM. Initial rates were recorded over the first 60 seconds of the reaction and analyzed using non-linear regression with the Michaelis-Menten equation, resulting in a well-fitted hyperbolic curve.

For kinetic data analysis, the Michaelis-Menten equation (equation 3) was employed, and non-linear regression of V₀ values as a function of substrate concentration was performed. The resulting curve showed an R² = 0.99, allowing for the determination of the maximum velocity (*V*_*max*_ ) and the Michaelis-Menten constant (*K*_*m*_ ) of the reaction at 23.03 µM/min and 18.80 µM, respectively, as shown in Figure 7.

**Figure 8:**
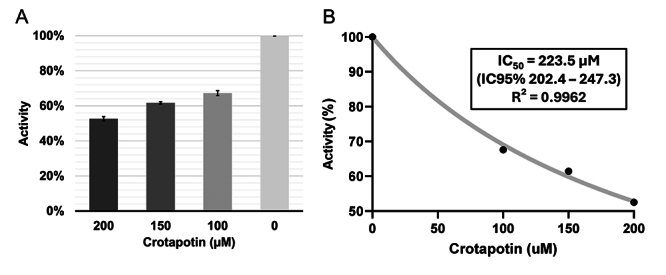
Inhibition assay of crotapotin on the activity of recombinant trypanothione reductase from *Leishmania braziliensis* (LbTR). **(A)** Percentage of enzymatic activity (Atv%) observed at different concentrations of crotapotin (0, 100, 150, and 200 μM), indicating dose-dependent inhibition. **(B)** F**.** itting curve for IC₅₀ determination, with an estimated value of 223.5 μM and a 95% confidence interval (CI95%) between 202.4 and 247.3 μM. The coefficient of determination, R² = 0.9962, suggests a good fit of the experimental data to the model.

The inhibitory activity of crotapotin against recombinant trypanothione reductase from *Leishmania braziliensis* (LbTR) was evaluated in microplate assays using different protein concentrations (0, 100, 150, and 200 μM). The results, presented in Figure 8, indicate partial and concentration-dependent inhibition of enzymatic activity, with a progressive reduction in residual LbTR activity as the concentration of crotapotin increases. Dose-response analysis revealed an IC₅₀ of 223.5 μM (CI95%: 202.4 - 247.3), indicating that relatively high concentrations of crotapotin are required to achieve 50% inhibition of LbTR. The curve fitting showed a determination coefficient (R²) of 0.9962, demonstrating the reliability of the data obtained.

These results suggest that, although crotapotin exhibits inhibitory potential against LbTR in *in silico* assays, this inhibition is limited in an *in vitro* system, requiring a high concentration to observe a significant effect. The moderate inhibitory efficacy of crotapotin suggests a low affinity for the target enzyme, possibly due to the extension of the TR active site and the molecular characteristics of crotapotin, which may restrict interaction with the active site of LbTR.

## DISCUSSION

### *In silico* inhibition assays

The enzyme trypanothione reductase (TR) is a widely recognized therapeutic target in combating *Leishmania* infections, being essential for the parasite’s metabolism and survival in hostile environments. The importance of TR lies in its crucial role in maintaining the redox balance within the parasite, a vital function for neutralizing oxidative stress generated by the host’s immune response. This defense mechanism allows the parasite to resist reactive oxygen species (ROS) produced by macrophages during infection, which utilize L-arginine to generate these ROS. In contrast, *Leishmania* competes for the same L-arginine to sustain the polyamine pathway, essential for TR production [69].

Given its critical function, TR has been the focus of investigations for the development of specific inhibitors that, in addition to targeting *Leishmania*, may exhibit low toxicity for the human host. Several studies have explored the efficacy of natural and synthetic compounds as TR inhibitors in various *Leishmania* species. This history of research and the validation of TR as a therapeutic target are supported by recent studies analyzing the action of different compounds in *in silico* and *in vitro* assays.

Studies by Battista et al. [35] demonstrated that structural optimization can enhance the efficacy and specificity of TR inhibitors, as observed with compounds derived from 5-nitrothiophene-2-carboxamides, which exhibited antileishmanial activity at micromolar concentrations. This finding supports the idea that modifications to the molecular structure of crotapotin could result in more effective interactions with TR [35]. Similarly, Sarfraz et al. [70] used virtual screening with the ZINC database to identify potential inhibitors of *Leishmania major* TR, highlighting the efficiency of the *in silico* approach for identifying promising molecules. When applied to crotapotin, this methodology offers new avenues to improve molecular affinity and stability [70].

In line with these studies, Maamri et al. [71] identified 3-methoxycarpachromene and masticadienoic acid as promising TR inhibitors, with low binding energy values and stability in the complex, supporting the viability of natural compounds as potential inhibitors. Crotapotin, with its immunomodulatory properties and intrinsic structural stability, could be positioned as a promising candidate in this context [71].

Studies conducted so far indicate a high structural conservation among TRs from different Leishmania species, allowing findings obtained in one specific model to be extrapolated to other species. Martinez-Calvillo et al. [72] demonstrated this high homology, justifying the use of *L. infantum* and *T. brucei* models to investigate *L. braziliensis* TR. Thus, based on the crystallographic structures of *Leishmania infantum* (PDB ID 4ADW) and *Trypanosoma brucei* (PDB ID 2WOW), it was possible to construct a reliable model of LbTR, enabling docking and molecular dynamics assays that represent the active conformation of the enzyme and ensure the stability of the observed interactions.

In the computational model of LbTR, crotapotin demonstrated moderate affinity for the TR active site, a hydrophobic pocket that favors interaction with molecules from apolar regions. Crotapotin, due to its composition rich in acidic residues and seven disulfide bridges, exhibits structural stability that facilitates hydrophobic interactions. Molecular dynamics simulations of 200 ns, with clustering analysis using the GROMOS method, identified 12 clusters, with cluster 1 being the most stable (RMSD of 0.189 nm), suggesting a stable conformation in the LbTR-crotapotin complex.

These structural properties of crotapotin, such as its rigidity conferred by disulfide bridges, promote stable occupation of the hydrophobic site of LbTR, suggesting that it may serve as a basis for the development of effective inhibitors, particularly if rational modifications are made. As described by De Oliveira et al. [49], crotapotin has specific positions tolerant to amino acid substitutions, which could allow for the introduction of hydrophobic residues to improve its affinity for the active site of LbTR.

The study by Battista et al. [35] demonstrated that, although compounds show promising interactions in *in silico* studies, their *in vitro* efficacy may be limited. Similarly, Fiorillo et al. [73] and Gonzalez et al. [74] emphasize the potential of natural products as effective and stable inhibitors, supporting the relevance of using natural derivatives in TR inhibition research.

Thus, the present study contributes to the discussion on the application of proteins from *Crotalus durissus terrificus* venom as a basis for molecular inhibitors, with crotapotin assays against LbTR representing a step forward in understanding its properties as a potential antiparasitic agent, opening new perspectives for the development of derivative compounds for the treatment of *Leishmania* infections.

### Purification and identification of crotapotin

The mass spectrometric analysis provided a reliable identification of crotapotin, based on the m/z peak at 9412, consistent with the expected characteristics for this molecule. This m/z value suggests that the sample contains the protein with a theoretical molecular mass close to 9442.25 Da, corroborating structural data available in the PDB database, specifically under ID 3R0L, which highlights the crystal structure of the CACB complex of crotoxin and reinforces the structural stability of crotapotin [75]. The MALDI-TOF^2^ technique proved effective in detecting and validating the identity of crotapotin, especially with the use of calibration standards covering a mass range from 700 Da to 37 kDa, enhancing the accuracy of the data obtained.

Additionally, the N-terminal sequence of crotapotin was aligned and showed significant homology with peptide chain sequences of *C. d. terrificus*, contributing to the confirmation of the identity of the isolated protein. These data further support the structural validation of crotapotin. The identity analysis performed adds robustness and reliability for future applications of crotapotin in antiparasitic research.

### Recombinant expression, purification, and characterization of LbTR

The recombinant expression of LbTR in *E. coli* was successful, yielding 4.8 mg/L with a high degree of purity, as verified by SDS-PAGE. The Ellman reaction was employed to validate the inhibition assays against TR, allowing for the continuous reoxidation of the dihydrotrypanothione product (T[SH]₂) back to the trypanothione disulfide substrate (T[S]₂). This method maintains constant substrate concentrations, significantly reducing the amount required, and enables continuous assays in microplates for 60 minutes or more, using minimal substrate concentrations (<1 µM) [36, 75].

The recombinant protein exhibited a molecular mass of 54 kDa, consistent with theoretical values, and its enzymatic activity was confirmed through the Ellman reaction, demonstrating that LbTR retained its catalytic function. Krauth-Siegel et al. [76] expressed *L. donovani* TR using the pTEX-LdTR plasmid, finding physical, spectral, and kinetic properties similar to those of other pathogenic trypanosomatids.

### *In vitro* inhibition assays

Studies on the proteins from *Crotalus durissus terrificus* venom have primarily focused on molecules such as crotamine, phospholipase A2 (PLA_2_), and crotoxin, investigating their anti-*Leishmania* properties, especially in *in vitro* models [51, 77]. In specific studies, crotamine, for example, demonstrated an increase in the efficacy of Glucantime® against *L. amazonensis* amastigotes in *in silico*, *in vitro*, and *in vivo* systems when used at concentrations of 100 and 3.125 μg/mL for crotamine and 300 μg/mL for Glucantime® [78]. Katz et al. [47] also evaluated the activity of *C. d. terrificus* venom fractions in macrophages infected with *L. amazonensis*, observing that crotamine exhibited the strongest inhibitory effect on parasite growth (IC₅₀: 25.65±0.52 μg/mL), while convulxin had a weaker inhibitory effect (IC₅₀: 52.7±2.21 μg/mL). Passero et al. [45] compared the antileishmanial activity of three different crotaline venoms and found that *C. d. terrificus* venom showed greater anti-*Leishmania* activity (IC₅₀ of 4.70 ± 1.72 μg/mL) compared to *C. d. cascavella* and *C. d. collilineatus*.

Additionally, Barros et al. [52] analyzed the cytotoxicity of PLA_2_ and peptides from *C. d. terrificus* in peritoneal macrophages infected with *L. infantum chagasi*, with an IC₅₀ of 98 μg/mL for PLA_2_ and 16.98 μg/mL for the peptide. Crotoxin also demonstrated activity against *Leishmania*, activating the M1 response in murine macrophages infected with *L. amazonensis*, with an IC₅₀ of 22.86 µg/mL for promastigotes and intracellular amastigotes [46].

However, these studies did not investigate in detail the inhibitory potential of these proteins on trypanothione reductase (TR), a relevant therapeutic target against parasites such as *Leishmania braziliensis*. In contrast, crotapotin, the acidic subunit of crotoxin, has been studied almost exclusively for its anti-inflammatory and immunomodulatory properties [50, 51, 77]. There are no reports of its effect on specific enzymatic targets, such as TR, making this study a pioneer in providing initial data on the interaction of crotapotin with this enzyme, essential for redox balance in *Leishmania*. The findings of this study provide new information on the inhibitory capacity of crotapotin against LbTR in *in vitro* systems, expanding its application beyond the immunomodulatory role and paving the way for its use in the bioprospecting of new antiparasitic agents.

Regarding toxicity, no toxicity has been reported in published studies following its application in small animals, such as birds, rats, and rabbits [79], and in subsequent studies, such as in edema induced in rat paws [80-82], Wistar rat lungs [83], and peritoneal macrophages of rats [84].

These initial findings open new research perspectives for crotapotin, highlighting the importance of exploring less-studied proteins from *C. d. terrificus* venom as promising alternatives to more extensively researched molecules. This work represents a step forward in the characterization of crotapotin and provides a foundation for future investigations that may optimize its affinity and efficacy against the enzymatic target trypanothione reductase in combating parasitic infections.

## Conclusions

The *in silico* assays, including molecular modeling and molecular dynamics simulations, proved promising, indicating a stable interaction of crotapotin with the active sites of LbTR and suggesting a possible steric inhibition mechanism. However, the *in vitro* assays did not reflect the same success observed *in silico*, with an IC₅₀ of 223.5 μM.

These results highlight the importance and utility of computational approaches in predicting and initially analyzing molecular interactions, while also underscoring the discrepancies that can arise between theoretical simulations and real biological systems. The modest inhibition observed *in vitro* may be attributed to various limitations, such as the high concentration of crotapotin required to achieve a significant inhibitory effect and the simplified conditions of the purified enzymatic system, which may not fully capture the complexity of cellular environments. Additionally, the peptidic nature of crotapotin presents challenges related to administration, bioavailability, and stability *in vivo*, in contrast to the small molecules frequently investigated as TR inhibitors. Therefore, to overcome these limitations and fully explore the potential of crotapotin, structural modifications to increase its affinity and bioavailability, as well as further testing in more complex and realistic systems, are recommended.

This study reinforces the need to integrate computational and experimental methods in the development of new therapeutic agents, while maintaining a critical perspective on the limitations of models used for predicting *in vivo* efficacy. Future studies should address these issues to validate crotapotin as a viable candidate for the treatment of leishmaniasis.

### Abbreviations

CA: crotapotin; CB: PLA_2_ from *C. d. Terrificus*; CL: cutaneous leishmaniasis; FAD: flavin adenine dinucleotide; GR: glutathione reductase; LbTR: trypanothione reductase expressed from *Leishmania braziliensis*; NADPH: nicotinamide adenine dinucleotide phosphate; TR: trypanothione reductase; TS₂: oxidized trypanothione; VL: visceral leishmaniasis.

## Availability of data and materials 

 The main data generated or analyzed during this study are included in this article. Any additional data will be made available by the corresponding author upon reasonable request.
